# Supplementary material for: Bioinformatics approach for developing a minimum set of SNP markers for identification of temperate japonica rice varieties cultivated in Spain
Source: PLoS One. 2023 Jun 23;18(6):e0286839. doi: 10.1371/journal.pone.0286839 (PMC10289409; doi:10.1371/journal.pone.0286839)
Supplement: S8 Table — (DOCX) [file pone.0286839.s008.docx]

**Supplementary File 8. List of groups of redundant markers for discriminating 27 rice cultivars as derived from genotypes previously obtained.**

[[1]] "chr1_625555" "chr1_869532" "chr3_35379886" "chr8_1997614"

[[2]] "chr1_745242" "chr1_1124636"

[[3]] "chr1_1374260" "chr1_1509911" "chr1_1872199" "chr1_2250313"

[[4]] "chr1_1622355" "chr8_25496149" "chr12_21873121"

[[5]] "chr1_1879431" "chr1_2124430" "chr2_5640329" "chr2_5742941" "chr8_3373317"

[[6]] "chr1_2001601" "chr1_27669307" "chr2_30629687" "chr2_31499012"

"chr3_1506097" "chr3_1629774" "chr3_28446934" "chr4_19122171"

"chr6_16513886" "chr6_24131541" "chr7_26125810" "chr8_3745545"

"chr9_817333" "chr9_879021" "chr9_3747461" "chr9_15752786"

"chr9_17253171" "chr9_18263911" "chr9_18471348" "chr10_4877258"

"chr11_19109346" "chr11_19401257" "chr11_20490702" "chr12_2165402"

[[7]] "chr1_2502946" "chr1_2581824" "chr1_2877160"

[[8]] "chr1_3124541" "chr2_5253225" "chr3_8658768" "chr5_26591553"

"chr6_8760332" "chr6_25248369" "chr8_26290663" "chr10_20754635"

"chr12_21332025"

[[9]] "chr1_3488077" "chr2_25755382" "chr3_4632823" "chr4_5868205"

"chr4_6258065" "chr4_6376847" "chr8_3626605" "chr9_16003139"

[[10]] "chr1_3712727" "chr1_4724131"

[[11]] "chr1_3998599" "chr2_16126515" "chr3_30752398" "chr5_1259691"

"chr6_28126955" "chr6_28186260" "chr6_28623873" "chr10_1088426"

"chr10_2501865" "chr10_4006252" "chr10_4736465" "chr10_6006075"

"chr10_10156203" "chr10_10751928" "chr10_10922296" "chr10_11124139"

"chr10_11125090" "chr10_11372671" "chr11_7255786"

[[12]] "chr1_4126084" "chr1_4207288" "chr1_4325184" "chr1_4349423" "chr1_5121565"

[[13]] "chr1_5769340" "chr1_29248859" "chr1_29639184" "chr1_41693336" "chr1_41878476" "chr2_1261211" "chr2_10376754" "chr2_10630489" "chr2_13284456" "chr2_15661575" "chr2_29893995" "chr2_32500801" "chr2_32769113" "chr2_34386360" "chr2_34723920" "chr2_35406962" "chr3_9718984" "chr3_9983936" "chr3_10126084" "chr3_17999538" "chr3_18500293" "chr3_19125149" "chr3_20500343" "chr3_20875010" "chr3_21752773" "chr3_22124754" "chr3_26005366" "chr3_31627459" "chr4_5499622" "chr4_5627505" "chr4_11217663" "chr4_12995709" "chr4_20376473" "chr4_20501585" "chr4_21248238" "chr4_24756463" "chr5_7625355" "chr5_11363201" "chr5_15876681" "chr5_17377379" "chr5_18500824" "chr5_23004836" "chr5_23250145" "chr6_874738" "chr6_1364862" "chr6_1866541" "chr6_2496114" "chr6_3993902" "chr6_30259427" "chr6_30829138" "chr7_3539086" "chr8_360157" "chr8_23125340" "chr9_7105211" "chr9_9500732" "chr9_14127074" "chr9_14625044" "chr9_18611086" "chr9_18751632" "chr9_22370267" "chr10_13500002" "chr10_17249867" "chr10_17813303" "chr11_1378959" "chr11_1999756" "chr11_2454788" "chr11_2502066" "chr11_2750402" "chr11_15123344" "chr11_20875110" "chr11_21502878" "chr11_28952396" "chr12_3132268" "chr12_3903357" "chr12_7751923" "chr12_8878025" "chr12_24124980"

[[14]] "chr1_5881271" "chr1_39875933" "chr1_40012174" "chr1_40750852"

"chr2_567926" "chr2_876199" "chr2_2720687" "chr3_10374886"

"chr3_12375271" "chr3_12625121" "chr3_21474466" "chr3_23630813"

"chr3_26125747" "chr3_26627237" "chr3_26875788" "chr3_30374583"

"chr3_32125148" "chr3_33755084" "chr4_19000052" "chr4_34271632"

"chr5_21757272" "chr6_2986200" "chr6_28376216" "chr7_28629490"

"chr7_28869229" "chr7_29122962" "chr8_8499109" "chr8_21625698"

"chr8_24489187" "chr11_17123875" "chr12_27471420"

[[15]] "chr1_6126957" "chr6_20637286" "chr11_14857272"

[[16]] "chr1_6487514" "chr2_2757887" "chr2_6869484" "chr2_7125785"

"chr2_8247668" "chr3_25253476" "chr3_25498720" "chr3_34002976"

"chr3_34506449" "chr3_34751187" "chr3_35498990" "chr4_25893924"

"chr4_27240817" "chr4_28242697" "chr4_29359792" "chr4_29999639"

"chr4_31205238" "chr4_31253312" "chr4_31375729" "chr5_21250432"

"chr5_22376878" "chr5_24876481" "chr6_7124919" "chr7_6052114"

"chr7_6256933" "chr7_8284964" "chr7_18130974" "chr7_19880684"

"chr7_20396179" "chr7_20625627" "chr7_21125708" "chr7_26628164"

"chr7_29002317" "chr9_19621813" "chr9_20498847" "chr9_20980033"

"chr9_21143531" "chr10_20250489" "chr10_20375501" "chr10_20624829"

"chr10_21887252" "chr11_8625666" "chr11_11970621" "chr11_15618382"

"chr11_18851240"

[[17]] "chr1_6625628" "chr2_8125803" "chr7_26451988"

[[18]] "chr1_7005626" "chr9_499488" "chr9_625057" "chr9_2379976"

[[19]] "chr1_7143891" "chr1_7216289"

[[20]] "chr1_7627395" "chr1_7749419"

[[21]] "chr1_7916522" "chr4_24251971" "chr4_24359219"

[[22]] "chr1_9204445" "chr7_7188320"

[[23]] "chr1_10873103" "chr1_36746330" "chr11_941149"

[[24]] "chr1_11251546" "chr1_12033280"

[[25]] "chr1_11667961" "chr1_11865108" "chr1_12129185"

[[26]] "chr1_12237687" "chr1_19542961" "chr1_22497886" "chr1_23388174" "chr1_24875035" "chr1_33170538" "chr1_33884355" "chr2_31230603" "chr3_3415338" "chr4_27616815" "chr4_30876584" "chr4_31502761" "chr6_7878708" "chr6_23868608" "chr6_26006761" "chr7_3127070" "chr8_8758307" "chr9_13999212" "chr9_14250057" "chr10_15878055" "chr11_26375946" "chr11_27761678" "chr12_1362094" "chr12_7496382" "chr12_14749443" "chr12_26125164"

[[27]] "chr1_12774971" "chr1_14289483"

[[28]] "chr1_12978640" "chr1_13236777"

[[29]] "chr1_13996402" "chr1_14885675" "chr1_15621948" "chr1_16292735"

[[30]] "chr1_13997074" "chr1_19600492" "chr1_20609300" "chr1_21750725"

"chr1_24875714" "chr1_25876262" "chr7_890526"

[[31]] "chr1_14632780" "chr1_15256205" "chr1_16012290"

[[32]] "chr1_15448056" "chr1_15605628" "chr1_16363714" "chr1_16677158"

"chr1_18504110"

[[33]] "chr1_15887532" "chr1_16250355"

[[34]] "chr1_21120592" "chr7_6375823"

[[35]] "chr1_21229845" "chr6_22154796"

[[36]] "chr1_21336764" "chr1_21425431"

[[37]] "chr1_23244155" "chr1_34191367" "chr1_35000182" "chr1_35501113"

"chr2_8472715"

[[38]] "chr1_24127610" "chr3_36132875"

[[39]] "chr1_24372781" "chr1_28626425" "chr2_15864948" "chr2_17246629" "chr3_12123244" "chr3_17624884" "chr3_21125914" "chr3_22492682" "chr5_16997750" "chr5_17999568" "chr5_21000355" "chr6_13494607" "chr8_26011566" "chr8_27252619" "chr9_16502519" "chr12_1246766" "chr12_2879754" "chr12_3500978" "chr12_27251759"

[[40]] "chr1_24451451" "chr1_24752676"

[[41]] "chr1_25024779" "chr1_25505469" "chr1_26250542"

[[42]] "chr1_25764555" "chr2_11006721"

[[43]] "chr1_26152958" "chr1_26375603"

[[44]] "chr1_26644062" "chr11_9805392"

[[45]] "chr1_27756192" "chr2_9876283" "chr2_19378835" "chr2_21123989"

"chr3_2173253" "chr3_26748178" "chr3_30501441" "chr4_33375466"

"chr4_34375202" "chr8_9375816" "chr10_17622324" "chr11_25625509"

"chr12_2628295" "chr12_4755252"

[[46]] "chr1_28004262" "chr6_24750108" "chr8_4503322"

[[47]] "chr1_28907413" "chr2_3142180" "chr2_29010844" "chr3_3068251"

"chr6_21885159" "chr6_27084347" "chr7_28155045"

[[48]] "chr1_29304844" "chr2_10254805" "chr2_10754859" "chr2_12634949" "chr2_14857660" "chr2_15501068" "chr2_17508000" "chr2_23125449"

"chr3_9983594" "chr3_34875783" "chr5_17249336" "chr5_17876622"

"chr7_13750836" "chr7_23751707" "chr10_499028" "chr10_3127541"

"chr10_3237936" "chr10_3377446" "chr11_17627521" "chr12_2757785"

"chr12_3745318" "chr12_4880368"

[[49]]"chr1_30244278" "chr1_30499870" "chr1_31000992" "chr1_31246608"

[[50]] "chr1_31748075" "chr1_33493964"

[[51]]"chr1_31976580" "chr1_32125015" "chr1_32258654" "chr1_32751637" "chr1_33031484" "chr1_34009118"

[[52]] "chr1_32367409" "chr1_32499753" "chr1_35875120" "chr1_36868426" "chr2_19066705" "chr2_20123840" "chr2_26251145" "chr3_9011499" "chr3_9253832" "chr3_9513844" "chr3_9619172" "chr5_5998793" "chr5_25257135" "chr5_25381071" "chr6_21125565" "chr6_21762042" "chr6_23128284" "chr6_27877704" "chr8_752144" "chr8_4243710" "chr8_5125780" "chr10_14362574" "chr10_16500148" "chr12_22874132"

[[53]] "chr1_33615150" "chr3_6126887" "chr3_7000522" "chr3_7132377"

"chr3_7249421" "chr3_7503977" "chr3_7623662" "chr3_16625492"

"chr9_14374758" "chr12_16500785" "chr12_17000504" "chr12_19128440"

"chr12_21619283"

[[54]] "chr1_34375623" "chr1_34761989"

[[55]] "chr1_35909958" "chr1_36007846"

[[56]] "chr1_36501158" "chr1_36648704"

[[57]] "chr1_37125903" "chr1_38011958"

[[58]] "chr1_37312643" "chr1_37670926" "chr1_37873247"

[[59]] "chr1_38637243" "chr1_38640378" "chr1_39128858"

[[60]] "chr1_42482961" "chr1_42624441" "chr1_43127459" chr3_10618542"

"chr3_10876758" "chr3_11000042" "chr3_11250408" "chr3_11625084"

"chr3_11751293" "chr3_11866840" "chr3_22578012" "chr3_23249980"

"chr3_34259275" "chr5_1126253" "chr6_4257821" "chr6_12869165"

"chr6_14133172" "chr6_14237444" "chr6_15377164" "chr6_16322857"

"chr6_16381437" "chr6_17622325" "chr6_20062811" "chr7_21244237"

"chr7_22499207" "chr7_23125701" "chr8_22220678" "chr8_28123931"

"chr11_8520909" "chr11_9000988" "chr11_17748964" "chr11_18377578"

"chr11_18615880"

[[61]] "chr2_353474" "chr2_762967" "chr2_978416" "chr2_1129600"

"chr2_1839730" "chr2_2015463" "chr4_30332382" "chr8_22002403"

[[62]] "chr2_1599527" "chr12_23246816"

[[63]] "chr2_3876275" "chr2_4377579" "chr2_4496203" "chr2_4869687"

[[64]] "chr2_4023667" "chr3_22758427"

[[65]] "chr2_5400526" "chr2_5476943"

[[66]] "chr2_7700999" "chr2_7879224"

[[67]] "chr2_8998512" "chr2_9010755"

[[68]] "chr2_10826432" "chr2_12005700" "chr2_12047115" "chr2_13132063"

[[69]] "chr2_11893523" "chr2_13005491"

[[70]] "chr2_17374207" "chr2_17607297" "chr2_18220317" "chr2_18241961"

"chr2_18509056" "chr2_18626835" "chr2_18699504" "chr2_19250583"

[[71]] "chr2_21002714" "chr2_21360023"

[[72]] "chr2_21877139" "chr2_23125560" "chr2_23875936" "chr2_24748616"

"chr2_26905644" "chr2_28010473" "chr3_7132605" "chr9_11125293"

"chr9_12375299" "chr9_12876580" "chr9_13377165" "chr9_16747377"

"chr9_16875877" "chr12_6922654"

[[73]] "chr2_22375014" "chr2_25232886"

[[74]] "chr2_23659072" "chr2_24498398" "chr2_25625688"

[[75]] "chr2_23749543" "chr2_25529282"

[[76]] "chr2_24876445" "chr2_25126832" "chr2_26002646" "chr3_5506710"

"chr3_5874917" "chr3_6639548" "chr3_8003273" "chr12_24248469"

[[77]] "chr2_26129292" "chr2_26130826"

[[78]] "chr2_26379603" "chr2_26620079" "chr2_27131419"

[[79]] "chr2_30098026" "chr2_30225675" "chr9_4502380"

[[80]] "chr2_32393228" "chr10_5874192" "chr10_10503656" chr10_10629728"

[[81]] "chr2_33183081" "chr2_33422315"

[[82]] "chr2_33866923" "chr2_34402176" "chr2_35478615"

[[83]] "chr2_35006159" "chr2_35122567"

[[84]] "chr2_35625606" "chr7_18681893" "chr12_22124895" chr12_22751225"

[[85]] "chr3_380530" "chr3_876546"

[[86]] "chr3_625735" "chr10_12382970" "chr10_13625278"

[[87]] "chr3_2374945" "chr8_7999612"

[[88]] "chr3_2877997" "chr4_22738479"

[[89]] "chr3_3624517" "chr3_3671198" "chr10_6485927" "chr10_6498163"

"chr10_8759467" "chr10_9750669" "chr10_12003343"

[[90]] "chr3_3875656" "chr3_3999437" "chr3_4127766" "chr3_4374343"

[[91]] "chr3_5999239" "chr3_6451980"

[[92]] "chr3_10248832" "chr12_7313135"

[[93]] "chr3_11088482" "chr3_11155290"

[[94]] "chr3_12249305" "chr3_12251328"

[[95]] "chr3_13453571" "chr3_13625054"

[[96]] "chr3_14873371" "chr3_15124181"

[[97]] "chr3_15746903" "chr3_15878375"

[[98]] "chr3_17003439" "chr3_17699030" "chr3_17863054" "chr3_20372180"

"chr8_24875371"

[[99]] "chr3_17125309" "chr3_18095781" "chr3_19004964" "chr3_19250700"

"chr3_20258172" "chr3_20375966" "chr3_20749529"

[[100]] "chr3_18441512" "chr3_18625371"

[[101]] "chr3_21369107" "chr8_24119932"

[[102]] "chr3_22644669" "chr6_17744823" "chr11_5498654" "chr11_11395651"

[[103]] "chr3_22999306" "chr3_23115600"

[[104]] "chr3_24009795" "chr3_24177311"

[[105]] "chr3_25751568" "chr3_26499375"

[[106]] "chr3_27125806" "chr3_29376353" "chr3_29987079"

[[107]] "chr3_27380054" "chr3_28120272" "chr3_29146373"

[[108]] "chr3_33261333" "chr3_33627587"

[[109]] "chr4_883602" "chr4_999534" "chr4_999864"

[[110]] "chr4_1126746" "chr4_1644548" "chr4_5250678" "chr5_3250079"

"chr5_3754821" "chr7_253659" "chr7_17875572" "chr9_2000226"

"chr9_3488060" "chr9_5121999" "chr9_6250907" "chr9_9249986"

"chr9_11374590" "chr11_21249738"

[[111]] "chr4_1247095" "chr5_8286883" "chr5_9242226"

[[112]] "chr4_1997408" "chr4_2033649"

[[113]] "chr4_3868941" "chr4_4242876"

[[114]] "chr4_4499277" "chr4_21121986"

[[115]] "chr4_6719609" "chr4_10750798" "chr4_11660460" "chr6_192409"

"chr9_22743171"

[[116]] "chr4_6876808" "chr4_8873599" "chr4_9003998" "chr4_9004145"

"chr4_9667682" "chr4_11502227" "chr4_11987210"

[[117]] "chr4_7244298" "chr4_7858140"

[[118]] "chr4_8000907" "chr4_12501434"

[[119]] "chr4_8125009" "chr4_11032629"

[[120]] "chr4_12373155" "chr10_1871964"

[[121]] "chr4_13771438" "chr4_16499136"

[[122]] "chr4_13970899" "chr4_14128286"

[[123]] "chr4_15543143" "chr4_17250626"

[[124]] "chr4_16625276" "chr4_17624751"

[[125]] "chr4_17004158" "chr4_17250285" "chr4_17499291" "chr4_17875212"

[[126]] "chr4_18028348" "chr4_18250186" "chr4_18373061" "chr4_18506511"

[[127]] "chr4_18749424" "chr4_23001296"

[[128]] "chr4_19374985" "chr4_19492146"

[[129]] "chr4_20228622" "chr4_20791853" "chr4_20879327"

[[130]] "chr4_21874415" "chr6_7615813" "chr6_7661762"

[[131]] "chr4_22112959" "chr11_4253060"

[[132]] "chr4_25061026" "chr4_25272508"

[[133]] "chr4_26385262" "chr4_26540394"

[[134]] "chr4_26869040" "chr4_27005551" "chr12_26171058"

[[135]] "chr4_27881803" "chr4_28190822"

[[136]] "chr4_28532916" "chr4_28776920"

[[137]] "chr4_29618783" "chr5_6628207" "chr6_7249825" "chr12_2252898"

[[138]] "chr4_30248673" "chr4_30497847" "chr5_2859034" "chr7_4197611"

[[139]] "chr5_1874063" "chr5_1999912"

[[140]] "chr5_2343600" "chr5_2372976"

[[141]] "chr5_2460569" "chr5_2753203"

[[142]] "chr5_5500521" "chr5_5621557" "chr5_5751682" "chr5_5876315"

[[143]] "chr5_6191511" "chr6_8122336"

[[144]] "chr5_6373236" "chr5_6375721"

[[145]] "chr5_7250058" "chr5_10976135"

[[146]] "chr5_7374776" "chr5_7375790" "chr7_23205173"

[[147]] "chr5_7500738" "chr5_7748249"

[[148]] "chr5_7909261" "chr5_10869400" "chr5_11250337" "chr5_11490649"

"chr5_13752083"

[[149]] "chr5_8250755" "chr5_8890508" "chr5_9124817"

[[150]] "chr5_8359966" "chr5_9000941"

[[151]] "chr5_9637802" "chr5_10643300" "chr5_10757848" "chr5_11894763"

"chr5_13752461"

[[152]] "chr5_12013821" "chr5_14251350" "chr8_27382304"

[[153]] "chr5_12628360" "chr5_13617349" "chr11_11121683"

[[154]] "chr5_14130484" "chr5_15000830" "chr5_15750085"

[[155]] "chr5_16251496" "chr5_16375040"

[[156]] "chr5_17749820" "chr5_17753488"

[[157]] "chr5_19125154" "chr5_19375044"

[[158]] "chr5_20125768" "chr5_20500839"

[[159]] "chr5_22882725" "chr5_23500972"

[[160]] "chr5_22960114" "chr5_23636626"

[[161]] "chr5_23749558" "chr9_19337813"

[[162]] "chr5_24678371" "chr5_25874796" "chr5_28626704" "chr6_11509110" "chr6_11699865" "chr6_27007824" "chr10_20210792" "chr10_21628908" "chr10_22128982" "chr10_22749493" "chr11_6376288"

[[163]] "chr5_25628523" "chr5_26015789" "chr5_27000112"

[[164]] "chr5_26387918" "chr5_26730502"

[[165]] "chr5_26874886" "chr10_21124435" "chr10_21373206"

[[166]] "chr5_27375072" "chr5_27501965" "chr5_27748072" "chr5_27872920"

"chr5_28000509" "chr5_28378820" "chr8_1225029"

[[167]] "chr5_29250053" "chr5_29374960"

[[168]] "chr6_266460" "chr6_601895" "chr6_24999963" "chr6_25000292"

"chr6_25622229" "chr9_20009713" "chr9_20122220" "chr9_21270336"

[[169]] "chr6_1260920" "chr6_1503256"

[[170]] "chr6_3151858" "chr6_3271741"

[[171]] "chr6_4377618" "chr6_4701804"

[[172]] "chr6_4927214" "chr9_8511502" "chr9_8511949" "chr9_10950275"

"chr12_1096907"

[[173]] "chr6_5995059" "chr6_6515068"

[[174]] "chr6_9122578" "chr6_9125050" "chr8_20878987"

[[175]] "chr6_9662016" "chr6_9939876" "chr6_9998875" "chr6_10127290"

"chr6_10376407"

[[176]] "chr6_13355270" "chr6_13437357"

[[177]] "chr6_14612486" "chr6_15152441"

[[178]] "chr6_18012379" "chr6_19246473" "chr6_19377695"

[[179]] "chr6_19502230" "chr6_19677552" "chr6_19749788" "chr6_19880232"

"chr6_20247077"

[[180]] "chr6_20501434" "chr6_20750600" "chr6_20878702"

[[181]] "chr6_21246944" "chr10_17992901" "chr10_18000023"

[[182]] "chr6_21611561" "chr10_19620297" "chr11_5882697"

[[183]] "chr6_22249886" "chr6_24038596" "chr6_24376327" "chr10_1631412"

"chr11_2758671" "chr12_2503528"

[[184]] "chr6_23960981" "chr6_24249769" "chr6_24285721" "chr6_24610877"

[[185]] "chr6_24872423" "chr6_24880447"

[[186]] "chr6_25751833" "chr6_26135231"

[[187]] "chr6_29359050" "chr6_29499199"

[[188]] "chr6_29874985" "chr6_30126243"

[[189]] "chr6_30500072" "chr6_31000351"

[[190]] "chr6_31065873" "chr8_14742914" "chr9_12250888" "chr11_5124349"

"chr11_5375011" "chr11_5624838"

[[191]] "chr7_994639" "chr7_1628175"

[[192]] "chr7_2034438" "chr7_2248309"

[[193]] "chr7_5560631" "chr7_5888791"

[[194]] "chr7_5713202" "chr10_618366" "chr10_1500932"

[[195]] "chr7_9151207" "chr7_9495936" "chr7_9878660" "chr7_10004558"

"chr7_10122799" "chr7_10373784" "chr7_10617704" "chr7_11248185"

"chr7_11379543" "chr7_11499004" "chr7_11620953" "chr7_13247218"

"chr7_15753720" "chr7_15976449"

[[196]] "chr7_13128278" "chr7_15214168"

[[197]] "chr7_16622044" "chr7_16779495"

[[198]] "chr7_17510516" "chr7_18399248" "chr7_18624441"

[[199]] "chr7_19125987" "chr7_19509833" "chr7_20002449" "chr7_20004555"

[[200]] "chr7_19624629" "chr7_19746554"

[[201]] "chr7_21503634" "chr7_21759341"

[[202]] "chr7_21624569" "chr7_22126610" "chr7_22259240"

[[203]] "chr7_23999071" "chr7_24099598"

[[204]] "chr7_24126745" "chr7_25248061"

[[205]] "chr7_26375840" "chr7_27013540" "chr7_28044556" "chr7_28339553"

"chr8_250551"

[[206]] "chr7_29245334" "chr11_3248714" "chr11_3501455"

[[207]] "chr8_880404" "chr8_2127987" "chr8_10501649" "chr8_10752775"

"chr8_11750133" "chr8_12376210" "chr8_13625357" "chr8_14125671"

"chr8_15126250" "chr8_16880600" "chr8_17371583" "chr8_18874411"

"chr8_19246647" "chr8_19498498" "chr8_19874377"

[[208]] "chr8_4627125" "chr8_5625460" "chr8_6371708" "chr8_7499996"

[[209]] "chr8_10161833" "chr8_10350263" "chr8_10879121" "chr8_11380257"

"chr8_11878985" "chr8_11997793" "chr8_13499030" "chr8_15999762"

"chr8_16012822"

[[210]] "chr8_10621946" "chr8_11253316" "chr8_12250562" "chr8_12875974"

"chr8_13749702" "chr8_14249872" "chr8_15499210"

[[211]] "chr8_12130417" "chr8_20042238" "chr8_20219130"

[[212]] "chr8_13990791" "chr8_14602111" "chr8_14877637" "chr8_15000367"

"chr8_18587016"

[[213]] "chr8_15874330" "chr8_16751448" "chr8_17712821" "chr8_18498625"

[[214]] "chr8_16994216" "chr8_16995473"

[[215]] "chr8_21749730" "chr8_21758984"

[[216]] "chr8_26500454" "chr8_26750020" "chr10_22996061"

[[217]] "chr8_27750156" "chr8_27805424" "chr8_27934561"

[[218]] "chr9_1126340" "chr9_1376156" "chr9_1878983" "chr9_2500973" "chr9_3499210" [6] "chr9_4373919" "chr9_4998986"

[[219]] "chr9_2370923" "chr9_2645073" "chr9_4747187"

[[220]] "chr9_8620344" "chr9_8751384" "chr9_8873207"

[[221]] "chr9_13315874" "chr9_21549989"

[[222]] "chr9_14882135" "chr9_14909175" "chr9_15076460" "chr9_15124521"

"chr9_15509574"

[[223]] "chr9_17873238" "chr9_22258265" "chr10_18993997" "chr11_26947252"

[[224]] "chr10_1002023" "chr10_1740590"

[[225]] "chr10_2180778" "chr10_2250747" "chr10_4211065" "chr10_4614429"

[[226]] "chr10_3703623" "chr10_3746000" "chr10_4749818"

[[227]] "chr10_5249019" "chr10_5500190"

[[228]] "chr10_5374498" "chr10_5625444" "chr10_5756368" "chr10_6878322" "chr10_8500756" "chr10_9121021" "chr10_10356947"

[[229]] "chr10_8405466" "chr10_8630564" "chr10_8781961" "chr10_9628349"

[[230]] "chr10_16127481" "chr10_16377502"

[[231]] "chr10_18749877" "chr10_18875694"

[[232]] "chr10_19251395" "chr10_19376912"

[[233]] "chr10_19502783" "chr10_19749679"

[[234]] "chr10_20880992" "chr10_21006624"

[[235]] "chr11_2960303" "chr11_3053022"

[[236]] "chr11_4605944" "chr11_4755998"

[[237]] "chr11_10359459" "chr11_10862536"

[[238]] "chr11_16572677" "chr11_16748262" "chr11_16890420"

[[239]] "chr11_26499789" "chr11_27109342"

[[240]] "chr12_6128026" "chr12_6140897" "chr12_6992932"

[[241]] "chr12_8000814" "chr12_8247212" "chr12_8435326"

[[242]] "chr12_9255870" "chr12_9501522" "chr12_9627132"

[[243]] "chr12_10156377" "chr12_12432179" "chr12_14537412"

[[244]] "chr12_11000880" "chr12_12106717" "chr12_12753926" "chr12_13627859" "chr12_14127126"

[[245]] "chr12_11380391" "chr12_12877780" "chr12_13124117" "chr12_13247585" "chr12_13501894" "chr12_14108497"

[[246]] "chr12_11625293" "chr12_12626119" "chr12_13110679" "chr12_13875859" "chr12_15263618" "chr12_15756312" "chr12_17624486"

[[247]] "chr12_14256332" "chr12_14875024" "chr12_16197828" "chr12_17501814"

[[248]] "chr12_18743841" "chr12_18875160"

[[249]] "chr12_21752257" "chr12_22375863"
